# [18F]FDG-PET/CT Demonstration of Young Adult Onset Stills Disease Mimicking Lymphoma

**DOI:** 10.1259/bjrcr.20210195

**Published:** 2022-02-16

**Authors:** Ayah Adel Nawwar, Julie Searle, Katherine Hodby, Nikesh Dhiraj Chavda, Naim Qamhia, Iain Douglas Lyburn

**Affiliations:** 1 Cobalt Medical Charity, Cheltenham, UK; 2 University Hospitals Bristol and Weston, NHS Foundation Trust, Bristol, UK; 3 Clinical Oncology and Nuclear Medicine department, Cairo University, Cairo, Egypt; 4 Gloucestershire Hospitals NHS Foundation Trust, Cheltenham, UK; 5 North Bristol NHS Trust, Bristol, UK; 6 Cranfield University, Bedford, UK

## Abstract

Adult-onset Still’s disease (AOSD) is an inflammatory disease of unknown aetiology, with no specific diagnostic tests. A 17-year-old female presented with fevers, night sweats and weight loss. Markedly FDG avid lymphadenopathy, and diffuse marrow and splenic uptake were demonstrated on [18F]FDG-PET/CT, most suggestive of lymphoma. After extensive investigations, the working diagnosis of AOSD was made. Other conditions, such AOSD, in addition to lymphoproliferative disorders, should be considered in the differential diagnosis of widespread avid lymphadenopathy in a teenager/young adult

## Clinical presentation

A 17-year-old female presented with a 6 month history of weight loss, fever, and rash. On clinical examination, there were enlarged cervical, axillary and inguinal lymph nodes and hepatosplenomegaly.

## Investigations, Outcome Follow-up

**Figure 1.**
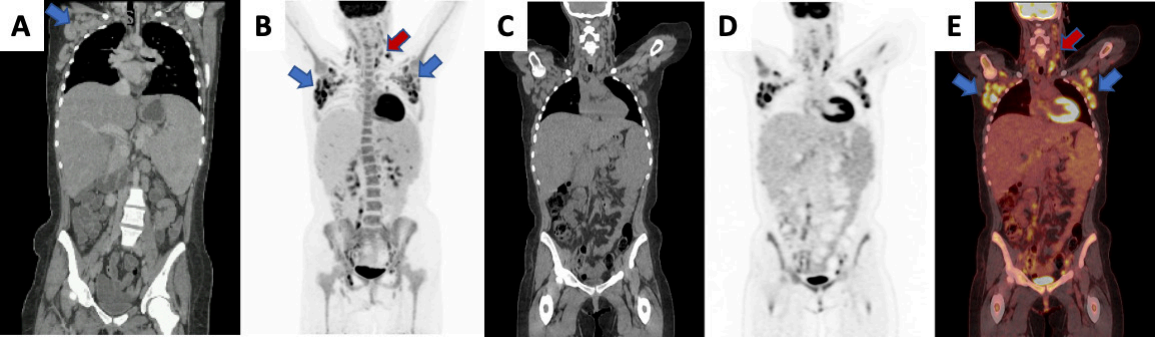
Initial CT (**A**) demonstrates lymphadenopathy above and below the diaphragm. Half body [18F]FDG PET/CT scan: Anterior Maximum Intensity Projection (MIP) (**B**) shows avid bilateral cervical/ supraclavicular (red arrows), axillary (blue arrows), retroperitoneal and iliac chains. Coronal images (**C-E**) demonstrate hepatosplenomegaly with elevated splenic uptake in relation to that of the liver (liver SUV max 2.2 and spleen SUV max 3.2) and diffuse increased marrow uptake. There is no abnormal cutaneous or periarticular uptake.

Haematological studies showed mild anaemia. C-reactive protein (CRP) and erythrocyte sedimentation rate (ESR) were elevated. Antinuclear antibodies (ANA) and rheumatoid factor (RF) were negative and tests for infection including salmonella and Lyme disease were unremarkable. On IV contrast CT, lymphadenopathy was found above and below the diaphragm (Figure 1*- A*), highly suggestive of a lymphoproliferative disorder.

[18F]FDG-PET/CT scanning was performed for further assessment, which demonstrated widespread avid lymphadenopathy, hepatosplenomegaly with increased splenic uptake in relation to that of the liver and diffuse increased marrow activity, Figure 1. *B-E*.

**Figure 2.**
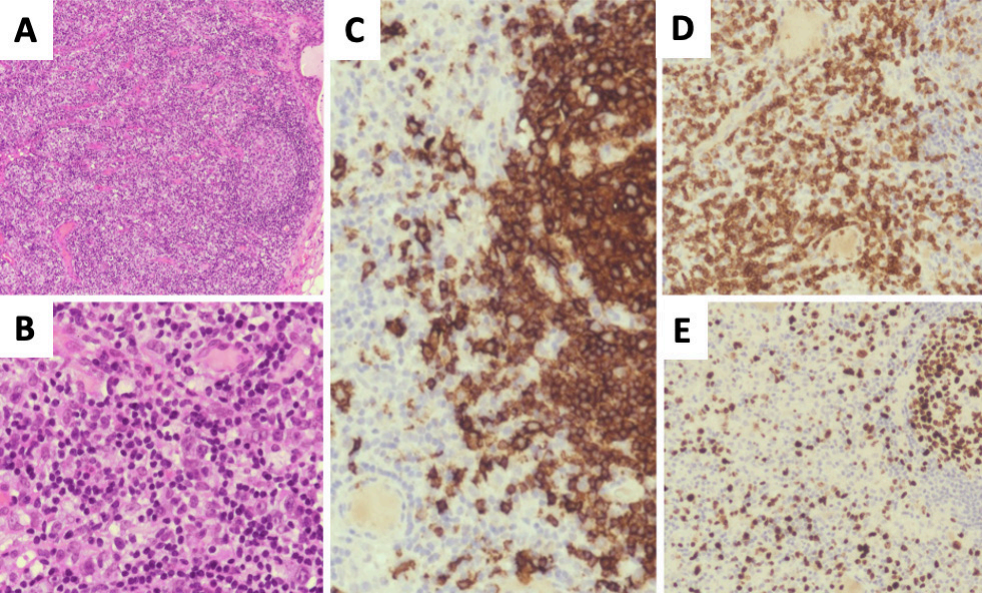
(**A**) x40 magnification H&E showing a lymphoid follicle with reactive germinal centre surrounded by expanded T-zone with prominent vascularity. (**B**) x200 magnification H&E showing eosinophils, plasma cells, scattered large lymphoid cells. (**C**) x1000 magnification- CD20 highlights the B cells in lymphoid follicle and some large lymphoid cells in the T-zone. (**D**) x1000 magnification-CD3 shows the expanded T-cell compartment with some large cells. (**E**) x1000 magnification-Ki67 highlights the reactive germinal centre and scattered large cells in the T-zone.

Repeated excisional nodal biopsies from the axillae demonstrated no evidence of clonal rearrangement of immunoglobulin (Ig) or T-cell receptor (TCR) genes to suggest a lymphoproliferative disorder, Figure 2. Features were more in keeping with a reactive process than a malignant lymphoproliferative disorder. Subsequently serum ferritin levels were measured and found to be highly elevated at 1, 545 ng ml^−1^.

Given the clinical picture of fever, organomegaly, anaemia and very high ferritin and reactive changes on histological analysis of the lymph nodes the working diagnosis of young AOSD was made. Symptoms markedly improved with oral prednisolone. She remains well at 9-month follow-up review with normal haemoglobin and ferritin just above normal levels

## Discussion

AOSD is a rare multisystemic connective tissue disease with many cases previously often ‘labelled’ as fever of unknown origin. AOSD usually affects young adults with fever, joint pain, sore throat and sometimes a distinctive salmon-coloured evanescent rash. Other observed clinical features include hepatomegaly, splenomegaly, lymphadenopathy and serositis. Patients may also experience the life-threatening complication of macrophage activation syndrome in up to 15% of cases.

The diagnosis remains challenging because clinical manifestations and radiological features overlap with a variety of other conditions, such as lymphoma and infections.^1^ Laboratory tests reflect the systemic inflammatory process showing high levels of erythrocyte sedimentation rate and C-reactive protein, however, ferritin levels are typically higher than with other disorders.^2^

Three established different clinical patterns of AOSD have been described: (1) monocyclic – single systemic episode; (2) polycyclic – multiple episodes lasting less than a year (alternating flares and remissions) and (3) chronic – persistent active disease and polyarthritis.^3^ The current management strategy is primarily symptomatic and largely empirical, aiming for symptomatic relief and prevention of organ damage and life-threating complications. The treatment for AOSD includes non-steroid antiinflammatory drugs, corticosteroids, and immune-suppressant agents such as methotrexate and azathioprine. Intravenous immunoglobulins may be used to treat an acute presentation; anti TNF-α and antiinterleukins have also been shown to be beneficial in treatment of refractory AOSD.^4^

AOSD largely remains a diagnosis of exclusion. To aid diagnosis, clinical criteria have been suggested by Yamaguchi.^5,6^ Yamaguchi’s major criteria include persistent fever, arthralgia/arthritis, non-pruritic salmon-coloured rash on trunk/extremities and granulocytic leucocytosis. Minor criteria include sore throat, lymphadenopathy, hepatomegaly or splenomegaly, abnormal liver function tests and negative RF and ANA tests. At least five features (including at least two major) are required for diagnosis. The patient presented had at least two major (fever and rash) and three minor criteria (lymphadenopathy, hepatosplenomegaly and negative RF/ANA tests).

Expert groups have attempted to define a specific pattern of uptake in [18F]FDG PET/CT which would suggest the diagnosis of AOSD. These features include widespread avid lymphadenopathy, elevated splenic uptake, diffuse bone marrow uptake, skin uptake corresponding to rash and periarticular uptake.^7–9^ Our case had avid lymph nodes above and below the diaphragm, elevated splenic uptake and diffuse marrow activity. [18F]FDG PET/CT can also help guide the site for lymph node or bone marrow biopsy, in addition to aiding diagnosis alongside the clinical features and laboratory findings.^4,5,10^

## Learning point

Although a lymphoproliferative disorder is the most likely diagnosis of widespread avid lymphadenopathy on [18F]FDG PET/CT, in a young adult AOSD should also be considered in cases when histological features are unclear and non-specific.
